# Study on the Impact of Historic District Built Environment and Its Influence on Residents’ Walking Trips: A Case Study of Zhangzhou Ancient City’s Historic District

**DOI:** 10.3390/ijerph17124367

**Published:** 2020-06-18

**Authors:** Fen Zeng, Zhenjiang Shen

**Affiliations:** International Joint SPSD Lab of Fuzhou University and Kanazawa University, Kanazawa 920-1192, Japan; zengfen186@163.com or

**Keywords:** built environment, walking behaviour, neighbourhood walkability, regeneration project

## Abstract

Walking maintains an indisputable advantage as a simple transport mode over short distances. Various situations have shown that when staying in a walk-friendly built environment, people are more likely to walk and interact with their surroundings. Scholars have reported some evidence of the influence of neighbourhood environments on personal walking trips. Most existing studies of the correlation between the built environment and walking, however, have been conducted in the West and are cross-sectional, which leaves a gap in addressing the causality between built environments and walking under the intervention of regeneration measures. This study takes a historic district of a mid-sized city in China as the research area and reports the changes in the traditional residential district’s built environment caused by the implementation of urban regeneration. In this paper, we use physical and perceptual indicators to measure the walkability of the built environment. We identify the changed content of the built environment’s walkability and the change of residents’ walking behaviour through longitudinal and quasi-longitudinal methods. The conclusion shows that the implementation of a regeneration project of the historic district has greatly changed perceived walkability, which has significantly promoted residents’ recreational walking trips, especially among the population of middle-aged and elderly people in the district. The conclusion that the built environment’s change promotes recreational walking is contrary to the research performed in sprawling Western contexts such as in the US, and it provides a meaningful supplement for research on the topic in an Asian context.

## 1. Introduction

Compared with other modes of transportation, walking is a very simple transport mode. It can improve personal physical activity, increase opportunities for informal contact, and promote neighbourhood relationships. Before the advent of major transformations in transport technology in the nineteenth century, walking was the common and traditional form of urban transport [1]. In contemporary society, especially within central areas, walking is also one of the fastest and most time-reliable transport modes for short-distance trips. However, car dependence with the development of large-scale urban motorisation has brought pervasive criticism upon unfriendly walking environments, such as single-function land, lack of service facilities, and poor sidewalk connectivity. This status is not conducive to public life, health, liveability, or economic improvement and could also frustrate pedestrians [2,3,4,5]. Consequently, researchers have started attaching importance to walkability, making the quality of the walking environment and the design of pedestrian precincts an essential element of urban planning in the past decade.

Reviewing previous research, there is enough evidence to prove a correlation between the built environment and walking behaviour [6,7,8]. The neighbourhood environment has been proven to at least partially affect individuals’ physical activity and walking behaviour [9,10,11,12,13,14,15]. Pedestrian activities can be increased by the presence of a well-connected street, easily accessible facilities, varied land uses, and good experience in the neighbourhood environment [16,17]. As one of New Zealand’s government entities, the New Zealand Transport Agency (NZTA) (2009) has offered an authoritative definition of walkability: “the extent to which the built environment is walking-friendly” [18]. In a pedestrian-friendly community environment, pedestrians prefer to interact with the surrounding environment more frequently, which is beneficial to creating a closer community network and a safer neighbourhood.

However, quantitative studies on the correlation between the built environment and walking have been focused on cross-sectional and easily raised causal attributions [19]. There is much less work focusing on longitudinal studies of built environments and walking behaviour under the influence of improvement measures. Besides this, on a neighbourhood environment scale, the sprawling Western context is different from the high-density and compact Asian context; those reports may not be applicable to Asia, including China [20]. Longitudinal research is helpful for improving the walking environment in China [21].

This paper provides new evidence for a longitudinal study of the relationship between the built environment and walking and helps to solve the problem of insufficient studies on this topic in Asian neighbourhood environments. The study measures “before” and “after” walkability of the built environment by indicators of three components and uses a quasi-longitudinal method to collect “before” and “after” information about residents’ walking behaviour to clarify the built environment’s impact on walking. This longitudinal report may help to provide some information for planners and managers who intend to optimise pedestrian environment quality to promote a more attractive, inclusive, and walking-oriented healthy city.

## 2. Literature Review

Reviewing the existing research, scholars have developed plentiful theoretical models to measure the extent to which the built environment is walking-friendly. Thus, the walkability index was generated [22,23]. The development of walkability measurement has gone through several stages. The earliest measurement indexes came from the 3Ds developed by Cervero and Kockelman (1997) [24]. Ewing et al. (2009) further extended this to a 5D layout, which included density, diversity, design, destination accessibility, and transit distance [25]. Frank (2010) calculated walkability by four walking indexes: intersection density, net residential density, retail floor area ratio, and land use mix [26]. All their measurement indexes are physical indexes. Pikora et al. (2003) and Cerin (2006) further developed the physical indexes, supplemented the measurement of aesthetics and pedestrian safety, and developed the theory of a neighbourhood environment walkability scale (NEWS) [27,28]. Jun and Hur (2015) emphasised perception and the walking experience in their model [29]. Moura (2017) adapted the 5C theory, which was developed by the London Planning Advisory Committee and proposed the latest 7C layout; “conspicuous” and “commitment” were added to the walkability indicators of “connected”, “convenient”, “comfortable”, and “convivial”, which makes the assessment more comprehensive within the expansion of the walking experience and perception [30]. Walking safety and experience indicators were also proved to be necessary and effective in measuring walkability [31].

In terms of types of research on correlations, scholars have provided multiple views to investigate the association between the built environment and walking, but the majority are cross-sectional. The existence of a small quantity of longitudinal studies generally concerns two categories: impact of event intervention or time sequence. The former refers to the implementation of an improvement plan or impact of a life event, such as a residential relocation or family or work changes, which involves a change of personal circumstances. The latter is based on the impact of different time periods at the same site. Regarding the impact of time, Hirsch et al. (2014) took five built environment factors as the measurement standard, inspected the same six locations in the United States four times over a decade, and found that the destination quantity and level of street connectivity were positively correlated with utilitarian walking. Rates of recreational walking increased under higher baseline levels of both lands zoned for retail and walking destinations but had no association with built environment features [32]. Regarding the impact of event intervention, based on changes in land use, bus support, pedestrian network, and population, which were caused by a natural experiment on Hong Kong’s university campus, Sun et al. (2014) measured the change of students’ walking behaviour. They found that the transformation of the campus environment led to a great change in students’ walking behaviour, and students’ walking distance and walking proportion were increased [33]. Carlson et al. (2019) studied the activity impact under a rapidly completed street-view improvement project in a northeast neighbourhood in Kansas City, MO. After recording “before” and “after” pedestrian activity at the same site, they found that the intervention with the street view increased pedestrian numbers [34]. Gao (2019) emphasised the impact of life-event induced change on walking. After collecting two-year travel records of 922 families in the Netherlands’ 87 cities, they analysed their walking behaviour and found that life events were related to utilitarian walking but had no significant impact on recreational walking [35]. The majority of this longitudinal research is based on the case of Western countries, and these reports do not consider factors that can influence pedestrian perceptions and experiences when measuring the extent to which the built environment supports walking. At a community level, the social network in a neighbourhood is more closely connected with the environment than in general areas [36,37,38,39,40,41,42]. As the majority of studies have been performed in Western countries, supplementation with Asian cases is urgently needed.

## 3. Study Area

### 3.1. District Selection

**Figure 1:**
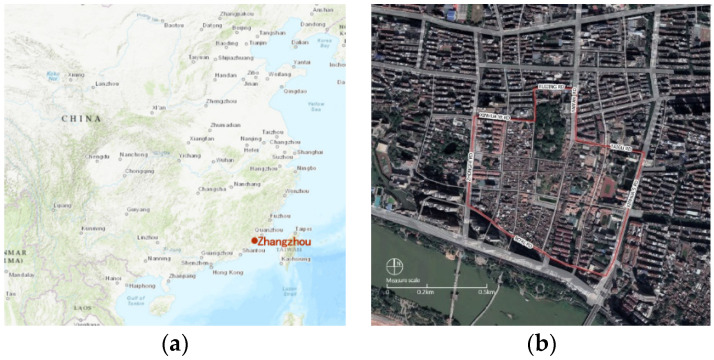
Location of study area: (**a**) Position of Zhangzhou municipality in China; (**b**) study area in Zhangzhou (2017 Google image).

The research area is the Zhangzhou ancient city’s historic district in Fujian Province, China, with an area of about 0.5 hm^2^ (Figure 1). According to the administrative division in Zhangzhou, the jurisdiction of the historic district belongs to the Xiqiao subdistrict, which includes four residential neighbourhoods. Although motorisation in Zhangzhou has developed rapidly in recent years, the main travel modes of this medium-sized city are still nonmotorised vehicles and walking (Table 1). The study’s neighbourhood is a traditional living settlement located in an old urban area, with a highly built footprint, low and continuous buildings, mixed land use and dense street networks. The existing studies’ summary concluded that within 500 m is a comfortable walking distance for most people. The plane morphology of the study neighbourhood is basically a structured grid square, and the straight-line distance from its geometric centre point to the boundary is at 300 to 500 m, which accords with the comfortable walking distance range, making it suitable for research.

### 3.2. Physical Intervention Descriptions of Phase I Regeneration Project

**Figure 2:**
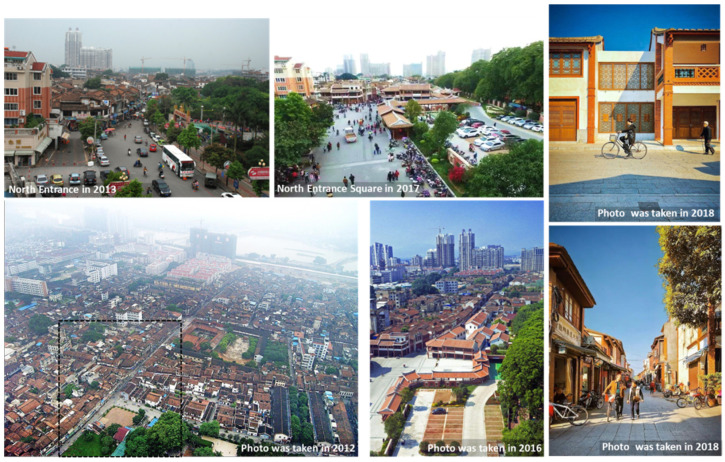
Photos of the historic district.

The ancient city’s historic district has implemented several local renovations since 1988, such as the restoration and renewal of local building facades and some street pavements. The first phase of the regeneration project, started in 2015, is a comprehensive, integrated vision and action for the entire historic district. It aims to improve the economic, social, and environmental conditions of the district and create a comprehensive community for life, culture, and tourism. The physical intervention of Phase I of the project mainly included traffic system optimisation, environmental design, commercial planning, and architectural renovation (Figure 2).

**Figure 3:**
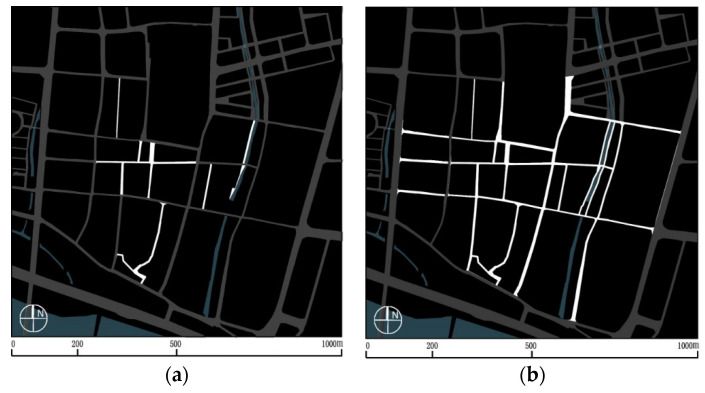
Neighbourhood traffic control (highlights show the streets are closed to motorised vehicles). (**a**) Before; (**b**) after.

The traffic system optimisation included an adjustment of the internal traffic mode and parking configuration. Previous vehicle lanes in the district were almost all adjusted to nonmotorised traffic use for pedestrian and nonmotor vehicle access (Figure 3). At the same time, the construction of an underground parking lot was carried out by using the idle land at the district boundary; a special entrance for vehicles and special connecting facilities for pedestrians were set up.

The environmental design includes improvement of district infrastructure and green space, allocation of street furniture, design of three entrance squares, and restoration of historic open space. The restoration of historic open space provides more leisure and exercise locations for residents and visitors.

Surrounding vacant lands were redesigned as commercial stores after the restoration of the historic open space, and some style conflicts and declining buildings were renovated or rebuilt.

## 4. Research Design and Methods

### 4.1. Analysis Model Construction

#### 4.1.1. Walkable Built Environment Model

This paper has summarised the development of measurement frameworks. As the physical calculation indexes were considered too incomprehensive to capture pedestrians’ walkability perception of the environment [30], the measurement index in this paper includes not only a developed physical environment index but also pedestrians’ perception and interaction indexes based on the characteristics of the built environment in the 7C index.

In the present case, we measured and collected 23 data sets to measure the “before” and “after” walkability. These data consist of three control components: Street Connectivity, Pedestrian Accessibility, and Perceived Walkability.

Street Connectivity. The measurement index was calculated by CAD based on the 1:2000 topographic file provided by the local government’s surveying department in 2012 and supplemented by the field survey.

Pedestrian Accessibility. The measurement index was an extracted survey of land use and destinations. The local government’s planning department’s status investigation files (before), delay images of Tencent and Baidu online street view (before), and the field survey (after) were aggregated to form the distribution of destinations and the land use calculation. Generally, residents’ pedestrian network in a neighbourhood incorporates formal and rich informal paths (in the study area, “informal paths” includes park paths that are used for transportation and other informal paths within the plot). Due to the accuracy of the topographic file, informal paths were able to be included in the calculation. As for the classification of destinations, according to the characteristics of traditional neighbourhood communities in Chinese cities, Yintao (2013) divided the neighbourhood destinations of Shanghai communities into 20 classes for measurement [44,45,46,47]. Alternatively, according to *The Shanghai Planning Guidance for 15-min Walkable Neighborhoods*, Weng et al. (2019) divided these into six categories and 15 subcategories, including education, medical care, municipal administration, finance and telecommunication, commercial service, and elderly care [45]. Considering the whole population of all ages, destinations were divided into four categories and nine subdivisions in this research. Table 2 shows the classification results of the trip destination statistics.

Perceived Walkability. Perception represents the extent to which people feel comfortable and safe walking. Although individuals have varied perceptions, there are also commonalities that can be tracked. In this research, we combined subjective evaluation data and objective data calculation for measurement. The aforementioned topographic file was used for the CAD calculation; questionnaire sampling survey statistics were used for measurement of subjective evaluation results.

#### 4.1.2. Survey and Sampling

In this paper, a quasi-longitudinal design was used to resolve the difficulties associated with previous data. Respondents were asked about their walking experience before and after project implementation. This method is considered to be an effective way to improve causality between the environment and trip, and the control influence of variables like attitudes over time [18]. Different personal demographic characteristics, like age or income, always produce variable neighbourhood activities. Demographic information and walking experience were obtained by the questionnaire. The sampling survey was conducted with the help of subdistrict staff from 16 to 26 November 2018. We conducted random interviews with families of community residents to ensure that respondents were evenly distributed. Residents who relocated after project implementation were not eligible to participate, and all respondents were granted anonymous use of their data for academic purposes. Through the questionnaire, personal and family attributes were collected, including age, gender, education, career status, any children in the household, annual household income, and car and electric bicycle ownership.

Residents were also asked to review their walking experience, previous and present. Firstly, residents needed to report a self-assessment of the project implementation influence degree on the change of personal walking behaviour and a multiple response assessment of the subjective evaluation of destinations based on their improved or reduced accessibility. Secondly, information about walking behaviour was measured, including walking frequency per week before and after project implementation, “before” and “after” average single walking distance, and “before” and “after” walking frequency to destinations.

### 4.2. Calculation and Statistical Methods of Variables

#### 4.2.1. Index Calculation

Calculation methods of the index measuring built environment walkability are as follows:

Group 1: Street connectivity index. (1) Block density: Number of blocks per unit area, indirect data were used to detect street connectivity; (2) Average length of street segments: Average length between two adjacent street intersections; (3) Street network density: Street length (km) on neighbourhood unit area (km^2^); (4) Connected Node Ratio (CNR) [11]: The ratio of the number of street intersections to the number of street intersections plus the number of cul-de-sacs. A CNR less than 0.5 should be avoided as much as possible; (5) Link-Node Ratio (LNR) [11]: The ratio of the number of road sections connecting two nodes and the number of nodes. When the LNR is higher, the connectivity is better. On a block with good connectivity, the LNR value should be greater than 1.4.

Group 2: Pedestrian accessibility index. Average walking distance to the aforementioned nine destinations and degree of district’s mixed land use, as follows.

(1) Average walking distance: Calculates the average walking distance of the nine classes’ destinations. Because the research neighbourhood plan is regular and square, walking distance between the geometric centre and the boundary is within 300 to 500 m. Therefore, the distance is within the predetermined walking range, and the average shortest walking distance from the four community geometric centres to the nearest three of nine classes’ destinations in the neighbourhood is calculated as follows to measure the average walking distance to a destination; the formula being $D_{j}=\frac{\sum_{i=1}^{3}D_{i}}{3}$.

(In the formula, $D_{j}$ is the mean value of the shortest distance from each community geometric centre to the nearest three destinations of class *j*.)

(2) Degree of land use mix: Proportion of each class of land area of the following five categories within a unit area (km^2^): Residential, Retail commerce, Public facility, Road traffic, and Green open space. Shannon’s (1948) [40] information entropy theory was used for reference to express land-use structure and equilibrium degree by the results of entropy value (H) and equilibrium value (J), using the formula H = $-\overset{n}{\underset{i=1}{\sum }}p_{i}\ln p_{i}$, J = H/H_max_ = $-\overset{n}{\underset{i=1}{\sum }}p_{i}\ln p_{i}/\ln n$.

(In the formula, *p_i_* represents the proportion of class *i* land area, and *n* represents the calculated land number.)

Group 3: Perceived walkability index. (1) Ratio of street walkable area: the ratio of walkable area to total neighbourhood area; (2) Proportion of green open space: the ratio between the area of green open space and total neighbourhood area; (3) Streetscapes’ suitability and walking safety: Evaluation results of respondents (from 1 point being “very dissatisfied” to 5 points indicating “very satisfied”) are used to qualitatively measure the index before and after project implementation; (4) Landmark visibility: 0–2 points are used to measure the visibility of landmarks like historic sites, characteristic buildings, squares, and parks, where 1 point is given to street segments that have a view of a landmark, 2 points are given to a situation where landmarks are located on street segments; otherwise, 0 points are given; (5) Interaction degree: The ratio of street segments with communication space to all street segments. The communication space here refers to open shops, restaurants, activity rooms, and other buildings that can initiate an interaction at night; (6) Regulatory enforcement degree: the ratio of street segments that have implemented current pedestrian-friendly regulations to all street segments, such as street segments with vehicle control, pavement arrangements, or crossing guidance interventions.

Walkability score of the built environment: Seven indicators of the above three components were weighted to get the results by weight function of an adult’s utilitarian walking following Moura’s 7C layout. The formula being

Walkability score = [(0.17 * *Connected node ratio*) + (0.06 * *Equilibrium degree*) + (0.17 * *Suitability of streetscape*) + (0.17 * *Interaction degree*) + (0.11 * *Landmarks visibility*) + (0.22 * *Walking safety*) + (0.11 * *Regulatory enforcement degree*)

#### 4.2.2. Methods

Statistical analyses of built environment variables and the survey were conducted in SPSS 22, and the significance level was set at *p* < 0.05. Univariate analysis examined the basic distribution of data for the primary calculation of descriptive statistics. The analysis method consists of two steps. Firstly, we explored the built environment contexts before and after project implementation. A statistical test was used to compare the response distribution; a paired sample *t*-test examines whether significant changes have taken place before and after. Analysis of covariance (ANCOVA) was used to test the difference between the three groups’ variables’ effects on the built environment before and after project implementation.

Secondly, we take the self-assessment result of project implementation influence degree on the change in personal walking behaviour (self-assessment result) as a variable and use Spearman’s correlation analysis to examine the statistical correlation between the self-assessment results and demographic characteristics. The purpose is to enable respondents to actively exclude possible subjective walking changes, so as to confirm that changes are caused by the implementation of the regeneration project. A chi-square test was used to further determine the classes of demographic variables with different changes. A paired *t*-test and Mann–Whitney–Wilcoxon test were investigated, respectively, for variables with a normal distribution (valid skewness and kurtosis thresholds were set between −2 and 2) and non-normal distribution. After comparing the visit frequency to destinations, walking frequency, and walking distance, the final results were used to explain the effective intervention of the built environment change on residents’ walking trips.

## 5. Results

### 5.1. Calculation Result of Built Environment Walkability Variables

#### 5.1.1. Elementary Analysis and Assumptions

Table 3 shows the calculation results of built environment factors under the implementation of the regeneration project.

(1) Interpretation of street connectivity indicators. There were enough blocks (C1 = 52) in the district. Together with the average length of street segments (C2 = 0.13 kilometre, about 0.08 mile), these showed correspondence with the eligible dimension range (0.12–0.15 kilometre), which was put forward by Jacobs (1993) [21]. The value of CNR (C4 = 0.98) revealed that there were almost no cul-de-sacs in the district. Combined with the value of LNR (C5 = 1.51), it showed that the district was composed of many small blocks and intersections with various paths between each block and consequently formed desirable street network connectivity.
(2) Interpretation of pedestrian accessibility indicators. Research points out that mixed-use of land is the key component of walkability, is linked with health, traffic, and environmental consequences, and is convenient for people to access on foot [16]. The equilibrium degree (A11 = 0.91) was close to 1, indicating the high mixed degree of land use belonging to a high-density residential neighbourhood. The distances between four centres of the research district grid and each of the nine types of facilities were almost all within 0.5 km. According to the international standard walking speed, the range that adults can reach in 5 min is 0.25 miles (about 0.4 kilometre), which shows that the neighbourhood has ideal walking accessibility to facilities [1,2,36].
(3) Interpretation of perceived walkability. Benefitting from the large area of an urban historical park (about 0.05 square kilometres) on its north side, the historic district had a high ratio of green open space. However, the walkable area outside the park was not desirable, and the engagement of local authorities in the pedestrian environment (W7 = 0.25) was insufficient. Therefore, the residents’ evaluation of streetscape suitability and walking safety (W3 = 2.86, W4 = 2.30) tended to be negative (median = 3). A large number of street segments with visible landmarks can improve street attractiveness and differentiation. The visibility of landmarks (W5 = 0.58) in the study area was unsatisfactory; otherwise, the street interaction degree was acceptable because of the central location in the city.

#### 5.1.2. Differences Analysis of Built Environment Variables among Groups

Table 4 reports the differences between “before” and “after” built environment variables. Both the correlation (*p* = 0.000) and *t*-test result (*p* = 0.032) were significant, which indicates that there is a significance difference. An analysis of covariance was further conducted to examine the intensity among three groups’ indicators, which led to the significance change (Table 5 and Table 6). The result of the test of homoscedasticity (*F* = 3.307, *p* = 0.059 > 0.05) was not significant, and there was no interaction (*F* = 0.359, *p* = 0.704 > 0.05). After removing the interaction terms, the result of tests of between-subject effects revealed a significant difference in the effects of three groups on the change of “before” and “after” built environment variables (Table 5). From the result of pairwise comparisons, we can confirm that the perceived walkability group has a greater impact function than the street connectivity group and pedestrian accessibility group (Table 6).

After weighting seven indexes of three components corresponding to the 7C framework, the walkability score before project implementation was 59.3, which belongs to a moderately walkable district according to the Walkscore^®^ standard. The walkability score (WS = 81.2) was greatly improved after project implementation. Based on the results of differences among the three groups of built environment variables, it can be concluded that the improvement of the walkability score is mainly due to the improvement of perceived walkability. Based on the above analysis results, we put forward a preliminary hypothesis that the changed built environment walkability mainly consists of walking perception and experience in the environment, which may promote the occurrence of recreational walking.

### 5.2. Analysis of Respondents’ Walking Behaviour

#### 5.2.1. The Report of All Respondents’ Samples

Table 7 displays the population characteristics of respondents. Results showed that the samples were mainly composed of middle-aged and elderly people with a low education level. Those with a full-time job and self-employed and retired respondents were homogeneously distributed, and the family income was mostly middle level. (According to the comparison of citizens’ income distribution from the *National Bureau of Statistics* and the economic indicators of personal capita income from the *Zhangzhou Statistical Yearbook 2017*, individuals with an annual income of more than ¥120,000 are classified as high-income citizens in the Zhangzhou municipality.) More than half of the families had children living in the home, and the proportion of those with a household car was not high. The sample represents the typical residents of the Zhangzhou ancient city’s historic district, a long-standing residential area with the highest ageing population of all subdistricts of the city (according to subdistrict staff). Most modern young or middle-aged families with a high level of education separate from their elders and move from the historic district to newly-built middle or upscale residential areas in the city to live independently.

The walking frequency variable and the variable of visit frequency to destinations that reflected the walking behaviour of residents were investigated by measurement data. The walking distance variable was reported as seven grades: 0.4, 0.4–0.8, 0.8–1.2, 1.2–2, 2–3, 3–4, 4–5, and above 5 km. A paired *t*-test was investigated to report the correlation of walking behaviours between “before” and “after”. The coefficient of correlation was significant (*p* = 0.000); the results of “before” and “after” walking frequency and walking distance were significantly different. The frequency of walking per week (*M_t_* = −0.939) increased significantly after project implementation (Table 8).

#### 5.2.2. Subgroup Analysis Results

Self-assessment data was investigated to three levels: no impact, certain impact, and great impact. A Spearman correlation analysis was run to test for socio-demographic variables and self-assessment data. The self-assessment results of four population attribute categories (age group, education group, career group, and car group) showed differences (Table 9). After a further chi-square test, despite the insignificant result on career groups, results were confirmed according to the adjusted residual (AR) value and the crosstab proportion of each class factor in the other three groups.

Residents aged 50 to 59 and over 60 in the age sub-group were more inclined to the following conclusion than other age groups—project implementation had a greater impact on their walking behaviour. The same results could be found in the “less than high school” class of the education group and the “no car” class of the car group.

Based on the above analysis conclusion, a paired *t*-test was run on subgroups to obtain results for “before” and “after” walking frequency and the walking distance of 57 samples without a car, 41 middle-aged and elderly samples, and 38 samples with less than a high school education background. Due to a large difference in the report result of each sample, the variable of visit frequency to destinations did not meet the valid threshold of skewness and kurtosis. A Wilcoxon signed rank test was consequently used to test the variables of the three subgroups. Only significant variables were shown in the final statistical results (Table 10 and Table 11).

(1) All three sub-groups reported increased walking frequency and walking distance. The increase in walking distance was significantly longer in the “no car” subgroup (*M* = −0.404), followed by the middle-aged and elderly subgroup (*M* = −0.366). The increase in walking frequency was significantly higher in the “less than high school” education subgroup (*M* = −0.658), followed by the middle-aged and elderly subgroup (*M* = −0.634).
(2) The report on visit frequency to destinations revealed specific contents of significant differences in the pedestrian behaviour of residents before and after project implementation. Among the three destinations, only the differences in green open space have commonality. In summary, walking distance and travel frequency of middle-aged and elderly residents improved after project implementation, which is reflective of recreational walks to visit the green open space. Kim et al. (2014) [23] indicated that recreational walking is more sensitive to the walking environment. The difference between built environment factors, however, was the comprehensive result of three group variables: street connectivity, pedestrian accessibility, and perceived walkability. From the specific content of respondents’ walking behaviour changes and the significant difference results of perceived walkability changes, at least one result could be measured: the change in the built environment brought about by the project has a significant relationship with the recreational walking promotion of middle-aged and older residents.

## 6. Discussion and Conclusions

Based on the change in the built environment brought by the implementation of the regeneration project, this study focuses on and evaluates the correlation of residents’ walking trips before and after the change through a quasi-longitudinal method. We adopt comprehensive indicators to evaluate built environment walkability, including not only developed physical environment calculation indicators but also perceived walkability and interaction indicators in the 7C layout. The results show that the project has significantly improved perceived walkability in the environment, and residents have also reported a desirable walking safety and streetscape experience index. Although there may be some residents with individual constraints or other objective factors, results can still be obtained from this paper. As we assume the increase in recreational walking is significantly related to the implementation of this project, the preliminary assumption of the correlation between recreational walking and the built environment is valid. For utilitarian walking, only positive changes in residents’ visits to retail venues and restaurants were observed, while the other content did not receive a strong data response in this study.

The first phase project of the Zhangzhou ancient city’s historic district retained the original form of its streets and alleys, mainly implementing environmental design and traffic system optimisation. The design of entrance squares and arrangement of landscape increased many open activity spaces; the optimisation of the traffic system reduced nuisances from vehicle traffic and enlarged pedestrian space, broadening the visual field. Intervention in commercial areas and growth of the tourism industry have turned many monotonous household-daily-necessity retail stores into featured local restaurants and diversified commodity retail, which facilitates more visits. However, there are still some limitations to this study. Even though scholars have been committed to providing reports on walking behaviour changes in built environments and discovering reasons for the changes via longitudinal design, subjective motivation, and triggers for walking are complex and uncertain. In addition to individual constraints, trip purpose or preferences generally affect the choice of walking for a private trip. Besides this, the perceived indicators of BE, such as site quality, health, neighbourhood satisfaction, or social connectedness, also effect walking results. Especially for residents who are familiar with the surrounding neighbourhood environment, even the captured changes may only be temporary results. The sustainability of the positive impact is still unknown; sometimes, social activities are more influential than the built environment for residents’ pedestrian behaviour [21]. Another limitation can be found in the content of pedestrian perception. Certain impacts on walking behaviour are found among the content of perceived walkability factors, but the specific pedestrian environment, community awareness, and detail factors (such as the architectural visual impact on both sides, street furniture, and street scale) that affect visual quality are not further captured. 

Nevertheless, although objective and perceived environment measures have been proven to be important environmental correlates of walking in adults, most studies in this field are still conducted in the West [3]. The sprawling Western context is different from the compact Asian context; relevant research in China holds great significance, especially the evidence reported in this study that change of BE promotes the recreational walking of the middle-aged and elderly, which helps to improve their physical function, reduce the economic and social burden of NCDs, and improve individual quality of life [26].

The results support the hypothesis that changes in the built environment can effectively promote leisure walking behaviour, especially for the middle-aged and elderly. We believe this research has made a meaningful contribution to the literature on this topic. As our study is a case study that may involve the unique characteristics of a single city, this limits generalizability. More cross-sectional cases, combined with longitudinal exploration, need further research and the assembly of a catalogue. In addition, future research will need to take into account other trip modes like driving or cycling to contribute a comprehensive strategy of BE impact on travel behaviour.

## Figures and Tables

**Table 1:** References for travel modes of similar medium-sized cities in China.

City	Walking Ratio (%)	Electric Vehicles Ratio (%)	Bike Ratio (%)	Public Transportation Ratio (%)	Car Ratio (%)	Year
Zhangzhou	21.17	25.50	6.84	6.08	13.46	2013
Huzhou ^a^	24.20	33.70	5.00	4.40	26.10	2015
Ningpo ^b^	4.53	22.28	18.27	34.38	15.06	2008
Fuzhou ^c^	28.30	14.00	14.60	16.70	9.20	2008
Kunshan ^a^	16.20	28.40	6.20	13.70	29.10	2017

^a^ From Bi and Luo (2018) [4]. ^b^ From Teng and Chen (2009) [43]. ^c^ From Lin T (2012) [27].

**Table 2:** Classification and statistics of nine types of trip destinations.

Destination Category	Subdivision Rules	Abbreviations
Commercial service destination	Retail store	RS
	Restaurant	R
Public facility destination	School	S
	Amenity facility	A
	Culture and recreation facility	CR
	Clinic	C
Transit destination	Bus stop	BS
	Parking lot	P
Green open space	——	GS

**Table 3:** Calculation results of built environment variables.

Components		Built Environment Variable	Value (Before)	Value (After)
Street connectivity	C1	Block density	52	54
	C2	Average length of street segments	0.13	0.12
	C3	Density of street network	16.82	18.02
	C4	Connected node ratio (CNR)	0.98	1.00
	C5	Link-Node ratio (LNR)	1.51	1.56
Pedestrian accessibility	A1	Distance to RS	0.06	0.08
	A2	Distance to R	0.16	0.13
	A3	Distance to S	0.32	0.37
	A4	Distance to A	0.24	0.25
	A5	Distance to CR	0.45	0.45
	A6	Distance to C	0.29	0.29
	A7	Distance to BS	0.52	0.41
	A8	Distance to P	0.00	0.42
	A9	Distance to GS	0.38	0.24
	A10	Entropy	1.46	1.55
	A11	Equilibrium degree	0.91	0.96
Perceived walkability	W1	Ratio of street walkable area	0.05	0.11
	W2	Ratio of green open space	0.10	0.15
	W3	Suitability of streetscape	2.86	4.07
	W4	Walking safety	2.30	4.22
	W5	Landmark visibility	0.58	1.00
	W6	Interaction degree	0.67	0.81
	W7	Regulatory enforcement degree	0.25	0.62
Walkability score	**WS**		59.3	81.2

**Table 4:** Paired-samples test result of built environment walkability (before and after).

Built Environment Variable	Mean	SD	SE	*t*	Df	Sig. (2-Tailed)
Before	−0.221 ^a^	0.728	0.031	−2.3	21	0.032
After	−0.151 ^a^	0.72				

^a^ Result of log transformation.

**Table 5:** Tests of between-subject effects from ANCOVA.

Dependent Variable	Type III Sum of Squares	df	Mean Square	*F*	Sig.
Corrected Model	2923.003 ^a^	3	974.334	5883.707	0.000
Intercept	0.712	1	0.712	4.302	0.052
Three groups of built environment variable	1.328	2	0.664	4.009	0.035
Before-After walkability	2146.077	1	2146.077	12,959.501	0.000

^a^*R*-squared = 0.999 (adjusted *R*-squared = 0.999).

**Table 6:** Pairwise comparisons analysis among three groups from ANCOVA.

Group of Variable		MD	SE	Sig ^b^	95% Confidence Interval for Difference ^b^	
					Lower Bound	Upper Bound
Group1	Group2	0.038	0.057	0.514	−0.082	0.159
	Group3	−0.212 *	0.059	0.002	−0.336	−0.088
Group2	Group1	−0.038	0.057	0.514	−0.159	0.082
	Group3	−0.250 *	0.045	0.000	−0.345	−0.156
Group3	Group1	0.212 *	0.059	0.002	0.088	0.336
	Group2	0.250 *	0.045	0.000	0.156	0.345

Based on estimated marginal means. * The mean difference is significant at the 0.05 level; ^b^ adjustment for multiple comparisons: least significant difference (equivalent to no adjustments).

**Table 7:** Characterisation of surveyed residents and their families.

Personal Attributes		*N*	Frequency (%)	Family Attributes		*N*	Frequency (%)
Gender	Male	45	54.88	Family size	1	5	6.10
	Female	37	45.12		2–3	43	52.44
Age	18–29 years old	4	4.88		4 or more	34	41.46
	30–39 years old	16	19.51	Annual household income	Less than ¥30,000	4	4.88
	40–49 years old	21	25.61		¥30,000–60,000	35	42.68
	50–59 years old	18	21.95		¥60,000–120,000	34	41.46
	60 years old or older	23	28.05		More than ¥120,000	9	10.98
Education	Basic first or second stage	39	47.56	Presence of children	0	33	40.24
	Secondary education or high school	27	32.93		1	37	45.12
	University	16	19.51		2 or more	12	14.63
	Master and above	0	0.00	Availability of a car	0	57	69.51
Career status	Student	3	3.66		1 or more	25	30.49
	Full time job	21	25.61	Availability of electric bicycles	0	21	25.61
	Self-employed	26	31.71		1	46	56.10
	No job	4	4.88		2 or more	15	18.29
	Retired	28	34.15				

**Table 8:** “Before” and “after” paired-samples test results of residents’ walking behaviour.

Walking Behaviour Variable	Before		After		Paired-Samples Test			
	Mean	SD	Mean	SD	Mean	SE	*t*	sig
Walking frequency per week	7.35	3.567	8.29	3.977	−0.939	0.200	−4.70	0.000
Average single walking distance	3.12	0.908	3.63	1.083	−0.512	0.072	−7.11	0.000

**Table 9:** “Before” and “after” correlation analysis between attribute variables and self-assessment variables.

Aspects	Variables	Correlation Coefficients	Sig (2-Tailed)
Personal attributes	Gender	0.002	0.987
	Age	0.363	0.001
	Education	−0.311	0.004
	Career status	0.281	0.011
Family attributes	Family population	−0.105	0.349
	Annual household income	−0.164	0.142
	Presence of children	−0.097	0.385
	Availability of a car	−0.248	0.025
	Availability of electric bicycles	−0.15	0.177

**Table 10:** “Before” and “after” paired-samples test results of three sub-groups’ walking behaviours.

Group Branch	Variable	Before		After		Paired-Samples Test				
		Mean	SD	Mean	SD	Mean	SE	*t*	df	sig
Middle-aged and Elderly	Walking frequency	8.44	3.905	9.07	4.274	−0.634	0.246	−2.57	40	0.000
	Walking distance	3.17	1.052	3.66	1.146	−0.366	0.091	−4.03	40	0.000
No car	Walking frequency	7.44	3.784	8.05	4.121	−0.614	0.192	−3.20	56	0.002
	Walking distance	3.21	0.940	3.61	1.161	−0.404	0.086	−4.68	56	0.000
Less than high school	Walking frequency	8.13	4.408	8.79	4.515	−0.658	0.254	−2.56	37	0.015
	Walking distance	3.34	0.994	3.63	1.195	−0.289	0.092	−3.16	37	0.003

**Table 11:** “Before” and “after” Wilcoxon test results of three sub-groups’ walking visits to destinations.

Group Branch	Variable	Related Samples Wilcoxon Test					
		Mean	SD	SE	*z*	*N* *	sig
Middle-aged and Elderly	Walking frequency to GS	3.82	2.46	5.852	2.392	38	0.017
No car	Walking frequency to RS	3.90	2.45	4.623	2.271	51	0.023
	Walking frequency to R	2.08	1.46	5.690	2.460	38	0.014
	Walking frequency to GS	2.83	1.91	15.652	3.354	48	0.001
Less than high school	Walking frequency to R	1.86	1.28	4.500	2.333	22	0.020
	Walking frequency to GS	3.97	2.58	5.852	2.392	31	0.017

* Samples with zero destination visits or no changes were removed.
